# The Impact of Strenuous Group Physical Activity on Mood States, Personal Views, Body Composition, and Markers of Myocardial Damage in Overweight/Obese Adults: The “Step-by-Step Italy's Coast to Coast” Trek

**DOI:** 10.1155/2014/854129

**Published:** 2014-07-22

**Authors:** Claudia Mazzeschi, Natalia Piana, Daniela Capezzali, Antonella Mommi, Cristina Aiello, Michela Gatti, Giannermete Romani, Livia Buratta, Dalila Battistini, Giovanni Nasini, Elisa Reginato, Lorena Urbani, Chiara Pazzagli, Carla Ferri, Giuseppe Ambrosio, Pierpaolo De Feo

**Affiliations:** Healthy Lifestyle Institute, CURIAMO, University of Perugia, Via Giuseppe Bambagioni 19, 06126 Perugia, Italy

## Abstract

It is clinically relevant to understand whether it is safe to recommend to trained overweight/obese people long-distance treks and whether these experiences could have a negative psychological impact or become even dangerous exposing the trekkers to the risk of clinically silent myocardial damage. To answer these questions we have performed a quantitative/qualitative study comparing the changes in mood profiles, personal views, body composition, and plasma troponin levels of 40 overweight/obese subjects with those of 36 healthy normal weight subjects after the participation in a trek of 388 km from the Adriatic to the Tyrrhenian seas trek: the “Step by step…Italy's coast to coast”. The results of this study demonstrate that long-distance treks are a safe activity for trained overweight/obese people which should be recommended because they improve mood, health status, and the relationship of participants with themselves and with the regular practice of exercise with effects similar to those obtained by healthy normal weight subjects.

## 1. Introduction

It is unknown what is the impact of strenuous exercise on overweight or obese people and whether related changes in body weight might influence the mood profiles and the views on physical activity. Potentially, strenuous exercise, like that experienced to perform long-distance treks, might be a stressor agent with a potential negative impact on mood state, views on physical activity [1–3], and the health condition. In this regard, it has been shown that even healthy marathon runners or triathlon athletes during strenuous competitions might experience increments in plasma troponin concentrations suggesting silent myocardial injury [4, 5].

It is clinically relevant to understand whether it is safe to recommend to trained overweight/obese people long-distance treks and if these experiences could have a negative psychological impact or become even dangerous exposing the trekkers to the risk of clinically silent myocardial damage. At present, it is not possible to answer this question because there are no studies reporting the impact of strenuous physical activity on mood states, personal views, and markers of myocardial damage in overweight/obese persons. For these reasons, we have performed a quantitative/qualitative study comparing the changes in mood profiles, personal views, body composition, and plasma troponin levels of 40 overweight/obese subjects with those of 36 healthy normal weight subjects after the participation in a trek of 388 km from the Adriatic to the Tyrrhenian seas trek: the “Step-by-step…Italy's coast to coast.”

## 2. Materials and Methods

### 2.1. Sample Description

Over the first two weeks of May, 2010 and 2011, 76 subjects participated in the “Step-by-step…Italy's coast to coast,” a trek across central Italy, between the cities of Ancona (Adriatic Sea) and Talamone (Tyrrhenian sea) for a total of 388 km in 13 days.

The participants were divided into two groups on the basis of their body mass index (BMI, cut off 25 Kg/m^2^): normal weight (*n* = 36, BMI 22.1 ± 1.0) and overweight/obese (*n* = 40, BMI 29.3 ± 3.2) subjects. The mean age of the normal weight was 54.2 (SD = 15.3) and that of the overweight was 58.0 (SD = 9.5) with no difference (*t*(60) = 4.54, *P* = .185).

Normal weight participants were habitual long-distance trekkers, in an apparent healthy state with the exception of one subject treated for hypertension; among the 40 overweight/obese, *N* = 19 subjects were treated for hypertension and 13 were affected by uncomplicated type 2 diabetes mellitus.

All the subjects of the overweight/obese group were trained before the trek for at least 10 months following the methodology of the lifestyle intervention trial performed at the Healthy Lifestyle Institute of Perugia University (CURIAMO trial, Australian New Zealand Clinical Trials Registry, ACTRN12611000255987) and approved by the local Ethics Committee (CEAS Umbria Region, HREC number 1/10/1633). The intensive part of the lifestyle intervention program, of three months' duration, was conducted in seven steps that involved different qualified personnel, as described in detail [6]. Briefly, during the intervention, patients underwent (1) an initial medical examination, (2) an assessment by a psychologist, (3) an assessment by a dietician, (4) a physical examination by a specialist in sports medicine, (5) an individualized program consisting of 24 sessions (two per week) of structured indoor exercise, (6) eight sessions of group therapeutic education, and (7) Nordic walking activity combined with walking excursions during weekends. In the maintenance phase of the lifestyle intervention, in order to sustain and improve personal motivation and socialization, we propose to patients the challenge to train for a difficult task such as a long-distance trek.

*Organization of the Trek and Meals*. The “Step-by-step…Italy's coast to coast” is a scenic itinerary of 388 km (GPS measurement) linking the cities of Ancona and Talamone by signposted footpaths. The trek distance has been distributed in 13 days with daily walking distances of about 30 km (28–36 km) in order to allow participants to reach by foot the hotels, after 8–10 hours of daily walk. The daily walking activities were organized in continuous 50–55 minutes of walks followed by 5–10 minutes of resting periods, which were utilized to drink water, to eat some snacks and/or fruit (approximately 20% of daily caloric intake) and, in the case of type 2 DM subjects, to check their capillary blood glucose. Every day, at about half the walking distance, a resting interval of 30–45 minutes was finalized for a picnic lunch to consume approximately 30% of programmed daily caloric intake. The dinner was consumed in the hotels' restaurants, about 1-2 hours after the arrival, and included the remaining 50% of the calculated daily caloric intake. The caloric intake of the diet, monitored by means of daily food diaries, was individualized on the basis of the estimated participants' basal metabolism [7] and energy expenditure to cover daily distances, estimated using the formula: 0,9 Cal × km × kg BW or, in a subgroup of 21 subjects, directly measured by a physical activity monitor (Sense Wear Armband, Body Media Inc., USA). During the trek, a diet with nutrient composition (55–60% of calories CHO, 15% protein, and 25–30% fat) similar in both groups but different in caloric composition was prepared. For normal weight participants the caloric intake of the diet was isocaloric; for overweight/obese participants it was slightly hypocaloric (about 200–300 Cal deficit).

*Quantitative Measurements*. Height, body weight, body composition by bioimpedenziometry (Tanita SC-330, Japan), waist circumference, and resting blood pressure (average of 2 measurements in the supine position) of all participants were measured on the first and last days of the trek, in the fasting state and after early morning urination. On the first and last days of the trek, 2 mL of blood was withdrawn from a subgroup of 27 participants, immediately refrigerated, and stored for subsequent analysis of cardiac troponin I levels (Troponin I insert 105146B, Beckman Instruments, Inc. Brea, CA). During the trek, blood pressure and heart rate were measured in the sitting position before and after the daily walk. All changes in drug assumption during the trek were recorded.

On the evening of the first and last days of the trek, participants were assessed for mood status using POMS (profile of mood states) [8]. POMS is a rating scale aimed at assessing different mood states, typically consisting of 58 adjectives referring to sensations rated by the subject on a five-point Likert scale (0 = not at all; 4 = very much). The Italian validated version [9] with “at this moment” instructions was used in order to measure the overall mood states of participants before and after participation in the walking trip. The six emotional dimensions of mood were used as indicator of overall distress: tension-anxiety, depression-dejection, anger-hostility, fatigue-inertia, vigor-activity, confusion-bewilderment, and the total mood disturbance score (TMDS, calculated by subtracting vigor-activity subscale from the sum of the remaining five factors).

*Qualitative Data Collection*. On the days preceding the start and the end of the trek, participants were invited to narrate, write, and share in group their emotions, difficulties, fears, needs, and limits about key aspects of their strenuous physical activity. The groups were conducted by a doctor of pedagogic sciences, using the focus groups technique: people were stimulated to express and share their experience in different ways, by alternating individual self-reflection via self-writing, with discussions in microgroups and in plenary ones. For all writings, participants were guaranteed complete anonymity. The narrative approach, by using self-narration and self-writing, enabled participants to acquire an in-depth understanding of their experience and their values and to evaluate their self-esteem and self-perception and the awareness of their condition [10]. At the beginning of the journey, the following topics were proposed for writing and discussion: (1) why am I here and what are my expectations? (2) What are my barriers, fears, needs, and wishes related to this experience? At the end, the proposed topics were as follows: (1) what this experience has meant to me; (2) one word or image to define this walk; (3) a significant memory of the journey; (4) a discovery; (5) a change.

### 2.2. Statistical and Qualitative Analyses

A *t*-test for independent sample was performed on quantitative data to test differences among overweight/obese and normal weight participants at baseline and on the changes scores (Delta) between baseline and over the journey values. Levene test has been used to interpret the variance homogeneity. A *t*-test for matched sample was performed in order to test differences in the two groups before and after the trek. Analysis was conducted using SPSS version 21.0.

Qualitative content analysis was conducted on pre- and postparticipants' writings in order to acquire feedback on personal views of the overall sample about the coast-to-coast experience to better understand the personal view on the experience, to identify the qualitative effect of the experience and the possible mechanisms through which physical activity influences mood [3]. All writings underwent qualitative content analysis by the extraction of semantic units and their grouping into micro- and macrocategories, through a process of understanding and interpretation [11]. Saturation was considered achieved when at least ten narratives reported the same content [10]. The analysis of all the texts was performed at the end of the intervention independently by two experts (N.P., G.R.) and the following aspects were identified as particularly relevant: (1) well-being, new self-perception, self-esteem, and self-empowerment, as benefits of the walk; (2) the relationship with one's own body; (3) the natural environment and being in a group, as facilitators for overcoming the challenge; (4) changes in the perception of oneself; in the relationship with others; in health status; in the motivation for future lifestyle changes.

## 3. Results

### 3.1. Quantitative Data

The participants of the normal weight group attended the walking trip for a mean of 9.1 ± 3.1 days and covered a total mean walking distance of 274 ± 92 km. The participants of the overweight/obese group attended the walking trip for a mean of 10.3 ± 2.9 days and covered a total mean walking distance of 308 ± 88 km with no significant difference. One subject of the normal weight group and two subjects of the overweight/obese group did not walk for all the consecutive days but rested for two days because of foot vesicles. Sixteen subjects of the normal weight group and thirteen of the overweight/obese group did not complete the entire distance of trek but walked for a minimum of 7 consecutive days. Their decision to cover half of the distance was taken before the departure, for personal time restrictions.

At baseline the two groups did not differ in mood status (POMS subscales), while the overweight/obese group had greater body weight, waist circumference, fat mass, and systolic and diastolic blood pressure in comparison to normal controls (see Table 1). Descriptive values at the end of the trek for the two groups are reported in Table 2. The subjects of the normal weight group had significant changes (in terms of reduction) in BMI (*t*(29) = 2.38, *P* = .028), body weight (*t*(29) = 2.36, *P* = .025), waist circumference (*t*(29) = 5.44, *P* < .001), and percent fat mass (*t*(34) = 6.06, *P* < .001). Regarding the mood, tension (*t*(22) = 4.35, *P* < .001), depression (*t*(22) = 3.03, *P* = .006), anger (*t*(22) = 2.94, *P* = .008), vigor (*t*(22) = 3.37, *P* = .003), confusion (*t*(23) = 4.04, *P* < .001), and TMDS (*t*(22) = 3.66, *P* < .001) decreased significantly while fatigue (*t*(22) = 4.02, *P* < .001) increased significantly. No differences were found for diastolic (*t*(20) = 1.91, *P* = .070) and systolic blood pressure (*t*(34) = .842, *P* = .409) and for fatigue (*t*(23) = −1.73, *P* = .097). The participants of the overweight/obese group had significant changes (in terms of reduction) in BMI (*t*(44) = 5.78, *P* < .001), body weight (*t*(44) = 6.32, *P* < .000), waist circumference (*t*(42) = 6.23, *P* < .001), percent fat mass, % (*t*(44) = 5.76, *P* < .001), and diastolic blood pressure (*t*(43) = 5.27, *P* < .001). Similarly to the other group, tension (*t*(32) = 3.89, *P* < .001), depression (*t*(44) = 3.07, *P* = .004), anger (*t*(35) = 3.51, *P* < .000), vigor (*t*(35) = 3.03, *P* = .005), confusion (*t*(35) = 5.13, *P* < .001), and TMDS (*t*(32) = 4.85, *P* < .001) decreased while fatigue increased (*t*(32) = 3.64, *P* < .001) significantly. No differences were found for systolic blood pressure (*t*(34) = 1.46, *P* = .152) and for fatigue (*t*(35) = −1.89, *P* = .079).

The comparison of the two groups on the change scores (differences between baseline values and values at the end of the trek) showed that the obese group differed from the normal weight group only for a greater reduction in body weight and BMI (Table 3). Both groups lost fat mass; the overweight/obese group lost body fat mass at a rate of 6.1 grams for every km walked and the normal weight group at a rate of 4.7 grams for every km walked (*P* = NS).

Plasma troponin I levels, measured on the first and last days of the trek in a subgroup of 27 participants (9 subjects of the normal weight and 18 subjects of the overweight/obese group), were undetectable in all subjects at baseline and at the end of the trek (all values <0.01 ng/mL).

During the trek, in the two DM2 subjects treated with insulin, it was necessary to avoid hypoglycemic episodes to reduce the daily insulin units by 45% and 60%; 14 out of 19 subjects treated for hypertension reduced the antihypertensive drugs (30% less calcium-antagonists and 65% less diuretics) because of the progressive reduction of systolic and diastolic blood pressure.

### 3.2. Qualitative Data

The set of writings allows defining the “coast-to-coast” experience as entirely positive, having allowed participants to find serenity (“a sense of accomplishment and serenity” and “a sense of general well-being”), harmony and peace with oneself (“I felt in harmony with nature and with myself”), and good mood (“A great joy filled my heart and enriched it”). The walk has stimulated listening to oneself, reflection, and recognizing one's own feelings and discomforts, inducing an “interior reflective change”.

Walking for long distances for many consecutive days has induced an interior change and a progressive open-mindedness (“I remember the eyes of my traveling companion, enlightened by something strong and beautiful that he kept closed inside” and “What struck me most in this trip has been my interior change, my starting to be interested in others and in the environment, and not only in myself”).

Participants narrate to have found new trust in themselves and to have felt “satisfaction for reaching a difficult target,” to be able to walk such a long distance for such a long time (“I discovered to be able to walk for 30 Km! And even for more than that!”).

Self-esteem is increased, as well as the awareness that one can attain the most difficult goals:* “I was afraid not to make it, to feel small, awkward, alone. But I am here now, and have accomplished an enterprise that has changed a lot inside me. I reached the independence I was looking for, the awareness that alone I can… I can make, feel, live.”*

Determination and will power may let one face the most difficult challenges:* “I got involved to demonstrate myself how much I can still do with so much will power,” “Mind and will power lead much further than feet, pain, blisters. One walks anyway and reaches anywhere.” *Reaching the goal brings about enthusiasm, happiness, and exaltation:* “I'm enthusiastic I've realized the dream I made that morning with open eyes, knowing that impossible dreams can often become true.”*

One of the most significant findings in participants' writings is the relationship with one's own body: “*the discovery of its own pace,*” its capacity of adaptation to fatigue and its potentiality:* “I won my fear of physical pain,” “I discovered I could also walk on blisters.”* Its limits and reactions are recognized: “*I've learned not to overdo physically, to stop before collapse.*” Participants find again confidence in their own body, “*its extraordinary strength and endurance”;* they confront their own age and their own strength:* “It's good listening to my own body, to its needs, its thoughts.”*

The body gains strength, faces hardships with more force, and is not afraid of rain, mud, or cold.

(1) Nature represents a privileged environment to walk: 13 days “among fields, woods, and the countryside” enjoying* “the landscape, the colors, the scents*,*” “the times and rhythms of nature,”* a detachment from routine and from everyday life. The immersion into nature becomes the way to* “recognize myself, the others, and our very human nature”* and to think about oneself and about new projects to realize. Nature frees subjects from sad thoughts and worries and gives strong emotions of beauty and astonishment. Nature becomes a resource, and weather problems are easily overcome:* “I discovered I could walk in the rain without any discomfort.”*

(2) The group was a key factor for the success of the journey. What cannot be done alone is made in group:* “I discovered that 30 Km are done together, but not alone”* and* “In good company, nothing is impossible.”* From an initial feeling of loneliness and discomfort, the group becomes space for meeting, sharing, understanding, acquaintance, and friendship:* “I realized I needed to talk, to share with others, to listen to their stories.”* The group gives support:* “The fact that we always helped one-another, with great reciprocal care, made me feel that I will never be alone”* and* “I was particularly supported by some people, who gave me one more stimulus to keep going, by listening to me and understanding me in my life problems.”* The group teaches respect and tolerance for the others and their diversity; it is a* “laboratory to fight intolerance.”*

(3) The most significant memory of the journey is the strength of the group* “which can always give to those who need without asking anything in exchange” and “the miracle of the individual who becomes community is accomplished.”*

The last key aspects which emerged from the writings are related to change in the perception of oneself, in the relationship with others, in health status, and in the motivation to future lifestyle changes.

(4) The journey gave a different, slower pace to the passing of days. Many wish to* “keep going slower in life”* and to* “observe what can be seen and savor every moment, without waiting for the next.”* Some tell of having got back to the center of their own life and of their interests, with a* “sense of well-being and of awareness of how life can be lived.”*

(5) Reaching the goal has strengthened participants' character, has charged them with* “pure vital energy”*, and has made some feel* “capable of facing any other challenge”* and of* “getting up after falling, never giving up.”* The journey becomes a metaphor of life, where* “also more distant horizons become attainable”* and where* “a new way to face life”* is discovered.

(6) The relationship with others has changed; the experience of travelling in group has cured shyness and has made people smile more. The way has helped participants to empty themselves and to reflect on oneself:* “The physical and medical aspect of the walk has not been the most important after all. It has rather been the catalyst of a deeper phenomenon: throwing off the mask, being able to show one's true self, which is almost always better than what we tend to show of ourselves in everyday life.”*

(7) Participants feel themselves more open and available for others whom they no longer will value* “on the basis of simple appearance.”* For many, being able to relate with others represents an important discovery:* “I feel more open to new acquaintances in group activities.”*

(8) From this revitalization with oneself and with others, the purposes of a more structured change* “that continues in life*” are born. Many patients write they want to continue to walk once back at home or they want to start a sport.

(9) The weight reduction and the better control of the disease obtained thanks to the walk represent a strong incentive to change one's lifestyle:* “As a matter of fact I have eliminated night insulin (all insulin). It's a real great change!”*

(10) Eating habits have changed:* “I can eat really less and I don't need alcoholics, at lunch and at dinner as well.*” Hunger attacks are controlled more easily, and there is a generalized wish to* “take care of oneself, of one's body,”* and of one's disease whose care is understood as important:* “I was an uninformed patient, who used to minimize and hide his disease. Now, thanks to you, I am a person who knows his diabetes, accepts it, and lives peacefully with it.”*

## 4. Discussion

This is the first study to compare the effects of strenuous physical activity between trained normal weight and overweight/obese trekkers. Our results support the conclusion that walking for long distances and consecutive days is a safe activity which can be recommended to either trained normal weight or overweight/obese persons because it improves body composition, blood pressure, mood status, feelings, and personal views on exercise.

Regular aerobic exercise and, in particular, walking are recommended to healthy subjects and to overweight, obese, and type 2 diabetic subjects [12]. Several epidemiological and intervention studies demonstrate that beneficial effects of walking become significant over 150 minutes per week with a dose response relationship [13, 14]. For this reason lifestyle interventions for obesity and/or DM2 prevention or care promote regular walking as a major component of the behavioral change [13, 14]. The results of the present study demonstrate that walking for long distances and several consecutive days has a positive impact on the health and mood status and opinion views of trained obese/overweight persons similarly to normal weight individuals. The trained overweight/obese people thanks to the long-distance trek significantly lost body fat mass, at a rate of approximately 6 grams for every km walked, and those on drug therapy reduced the use of antihypertensive and antidiabetic drugs. We also demonstrate that the strenuous physical activity, about 30 km walked every day, does not increase the risk of silent cardiac damage, as assessed by measuring plasma troponin levels.

Regarding the changes in mood status, our findings are in agreement with previous studies showing the positive effects of physical activity on mood [15] and enlarge also the benefits of strenuous exercise also if performed by trained overweight/obese individuals. Engaging in regular physical activity displays better general health outcomes, higher values of health-related quality of life, and better mood states [16]. Specific to mood in the general population, there is a wealth of research which indicates that regular physical activity increases positive mood states (e.g., vigor, friendliness) and decreases negative mood states (e.g., depression, anger, and hostility) [16]. Many cross-sectional studies support the evidence that exercise, physical activity, and physical activity interventions have beneficial effects across different populations with different physical and psychological conditions on well-being [17]. In a review of literature, A. Byrne and D.G. Byrne [18] indicated that 90% of studies demonstrated a decrease in depression and anxiety after participation in physical activity interventions. Obesity is often associated with depression [19, 20]. Depressive mood is considered a cause of the attrition to physical exercise, and a main bulk of the research has been devoted to exploring the association between physical activity and depression or mood disorder in obesity [20–22]. In obesity regular exercise is both a predictor of weight loss and generally associated with improvements in mood profile [23]. Mood profile has been generally investigated by the profile of mood states (POMS) [8], a scale born in the context of counseling or psychotherapy and lately applied in sport and exercise contexts [3]. A review by Berger and Motl [3] has documented the use of POMS in exercise settings, supporting the relationship between exercise and acute mood changes. Our study demonstrates that the improvement of mood, documented by POMS, was similar in the groups of normal weight and overweight/obese, suggesting that when the participants are trained a greater body weight does not preclude the psychological beneficial effects of regular exercise.

Although the mechanisms through which physical activity influences mood are not completely explored [3, 19], the writings of the participants in our study allow identifying some facilitators which influence the mood during a strenuous physical activity. According to the opinion views of our overweight/obese participants, a central facilitator role is played by the opportunity to share their experience of obesity with others like themselves; the group served as a resource and a stimulus, as well as a means of escaping from loneliness and socializing. Another important facilitator is positive thinking, believing in the project with determination, being confident about one's abilities, and accepting the challenge in order to prove to oneself and to others that success can be achieved. The key is found in one's relationship with himself/herself. The participants thanks to the trek acknowledged the positive aspects of physical activity, describing it as an experience of pleasure, fun, and well-being, and asked to repeat it again. The participants report, in agreement with the quantitative data of the POMS, that the trek had positive psychological effects, made them feel free, reduced their stress levels, reinforced their self-confidence, and enhanced the harmony between body and mind.

In conclusion, long-distance treks are a safe activity for trained overweight/obese people which should be recommended because they improve mood, health status, and the relationship of participants with themselves and with the regular practice of exercise with effects similar to those obtained by healthy normal weight subjects. It should be pointed out that the study has concerned a cohort of obese people with no different baseline mood scores than normal weight people, perhaps thanks to the care and training program these people were offered before the trek and/or thanks to a sort of “natural selection” of participants operated by this pretrek program. Results may be different for cohorts of obese people with significantly different mood scores in respect to normal weight people.

## Figures and Tables

**Table 1:** Baseline characteristics of the two groups of participants to the trek and *t*-test results. Data are presented as mean ± SD.

	Normal weight (*n* = 36)	Overweight/obese (*n* = 40)	*t*	*P* value
BMI (kg/m^2^)	22.1 ± 1.0	29.3 ± 3.2	10.98	.000
Body weight (kg)	62.2 ± 9.2	83.3 ± 14.7	4.76	.000
Fat mass (kg)	14.5 ± 3.7	25.2 ± 6.6	4.08	.000
Waist circumference (cm)	82.1 ± 6.8	100.2 ± 9.5	11.12	.000
Systolic BP (mmHg)	118.1 ± 14.6	133.2 ± 14.8	3.15	.003
Diastolic BP (mmHg)	77.7 ± 8.8	83.0 ± 8.3	1.85	.007
Tension	6.76 ± 3.40	7.02 ± 5.81	.207	.837
Depression	4.96 ± 5.72	4.34 ± 5.47	−.13	.897
Anger	3.76 ± 4.02	6.40 ± 9.34	1.4	.167
Fatigue	18.48 ± 5.59	20.28 ± 5.26	1.23	.221
Vigor	4.20 ± 3.03	3.91 ± 4.36	−.087	.931
Confusion	6.64 ± 3.82	5.57 ± 4.35	−.778	.440
TMDS	−1.44 ± 2.94	−.91 ± 4.31	1.015	.314

**Table 2:** Descriptive statistics for body composition and physiological and mood values at the end of the trek for the two groups. Data are presented as mean ± SD.

	Normal weight	Overweight/obese
BMI (kg/m^2^)	21.81 ± 2.36	27.81 ± 2.51
Body weight (kg)	61.81 ± 8.38	80.73 ± 10.63
Waist circumference (cm)	78.14 ± 5.52	96.79 ± 8.39
Fat mass (%)	20.81 ± 4.89	27.76 ± 7.04
Systolic BP (mmHg)	119.29 ± 10.96	127.86 ± 12.04
Diastolic BP (mmHg)	71.43 ± 2.44	75.36 ± 6.92
Tension	2.71 ± 3.4	1.5 ± 1.78
Depression	.85 ± 1.86	1.14 ± 1.65
Anger	.14 ± .37	2.07 ± 3.14
Fatigue	22.57 ± 7.81	23.78 ± 3.88
Vigor	3.28 ± 3.25	2.28 ± 2.26
Confusion	3.00 ± 2.52	3.35 ± 3.18
TMDS	29.73 ± 12.48	30.17 ± 8.09

**Table 3:** Means and SD for changes (Delta) of the measured parameter in the two groups after the trek. Comparison between the normal weight and the overweight/obese groups.

Delta	Normal weight		Overweight/obese		*F*	*P*
	Mean	SD	Mean	SD		
BMI	.15	.36	.40	.47	6.37	.014
Body weight (kg)	.42	.96	1.06	1.13	6.61	.012
Waist circumference (cm)	1.29	1.27	1.59	1.67	.665	.417
Fat mass (%)	1.78	1.64	2.02	1.64	.237	.629
Systolic BP (mmHg)	2.59	14.34	3.06	12.34	.017	.897
Diastolic BP (mmHg)	3.91	9.36	6.14	6.89	1.05	.309
Tension	4.09	5.94	4.03	5.94	.001	.969
Depression	3.30	5.22	3.00	5.85	.041	.840
Anger	4.55	7.78	1.86	3.04	2.48	.121
Fatigue	−3.75	10.62	−1.75	5.81	.884	.351
Vigor	2.13	3.03	1.88	3.73	.067	.796
Confusion	3.41	4.13	2.69	3.15	.588	.446
TMDS	6.73	8.83	9.81	11.61	1.15	.288
